# Determination of the Accuracy of the Straight Bevel Gear Profiles by a Novel Optical Coaxial Multi-Ring Measurement Method

**DOI:** 10.3390/s23052654

**Published:** 2023-02-28

**Authors:** Junheng Li, Dehai Zhang, Yanqin Li, Xuanxiong Ma, Tao Wang, Chao Wu

**Affiliations:** 1 Mechanical and Electrical Engineering Institute, Zhengzhou University of Light Industry, Zhengzhou 450002, China; 2 Henan Key Laboratory of Intelligent Manufacturing of Mechanical Equipment, Zhengzhou 450002, China; 3 Energy and Power Engineering Institute, Zhengzhou University of Light Industry, Zhengzhou 450002, China

**Keywords:** straight bevel gear, surface profile error, measuring accuracy, coaxial multi-ring measurement method, machine vision

## Abstract

Straight bevel gears are widely used in mining equipment, ships, heavy industrial equipment, and other fields due to their high capacity and robust transmission. Accurate measurements are essential in order to determine the quality of bevel gears. We propose a method for measuring the accuracy of the top surface profile of the straight bevel gear teeth based on binocular visual technology, computer graphics, error theory, and statistical calculations. In our method, multiple measurement circles are established at equal intervals from the small end of the top surface of the gear tooth to the large end, and the coordinates of the intersection points of these circles with the tooth top edge lines of the gear teeth are extracted. The coordinates of these intersections are fitted to the top surface of the tooth based on NURBS surface theory. The surface profile error between the fitted top surface of the tooth and the designed surface is measured and determined based on the product use requirements, and if this is less than a given threshold, the product is acceptable. With a module of 5 and an eight-level precision, such as the straight bevel gear, the minimum surface profile error measured was −0.0026 mm. These results demonstrate that our method can be used to measure surface profile errors in the straight bevel gears, which will broaden the field of in-depth measurements for the straight bevel gears.

## 1. Introduction

The straight bevel gear is a very important mechanical transmission component that is used in mining, metallurgy, shipbuilding, automobile, and other industries due to its advantages, such as large overlap coefficients, large bearing capacity, and stable transmission. The geometry and manufacturing accuracy of the straight bevel gear teeth have a significant impact on their performance, longevity, and reliability, and hence measuring and evaluating the straight bevel gear products is essential to ensure their quality.

Measuring these errors and evaluating the accuracy of the straight bevel gears has long been a subject of high research value for scholars both at home and abroad, and many scientists have conducted research on this subject. Guienther et al. proposed a new measurement standard based on geometry for measuring bevel gears; although this could improve the machining quality of bevel gears, it was not as stable as it could be [1]. Zhang et al. proposed a method for measuring and evaluating the overall error based on the characteristics of spiral bevel gears [2]. Fu et al. combined laser plane generation technology with laser detection technology and embedded control theory. They proposed a new method for detecting the flatness of a spiral bevel gear online [3]. Liu et al. proposed an accurate compensation method for the inclination error in the tooth surface to improve the accuracy of measurement of spiral bevel gear tooth surfaces [4]. Li et al. utilized the DMAIC method of the six-sigma approach, involving definition (D), measurement (M), analysis (A), improvement (I), and control (C) to carry out quality control of spiral bevel gears [5]. By adjusting the parameters of the machine tool, gear cutter, and spiral bevel gear, Wan et al. determined the meshing point coordinates of the convex and concave surfaces of the spiral bevel gear. They proposed a measurement control strategy and a corresponding solution algorithm [6].

Kasumasa Kawasaki and colleagues used coordinate measurements to measure the pressure angle, tooth angle error, workpiece installation angle, etc., of gear products manufactured on CNC milling machines. However, they reported that improving the machining accuracy of end milling machine gears remained a challenge [7]. Li et al. proposed a new method for achieving accurate measurement and evaluation of the machining error for spur bevel gears, which involved extracting the three-dimensional (3D) model surface designed by the manufacturer as a reference to complete the measurement to ensure the correctness of the error result [8]. Li et al. used machine vision to obtain an image of a logarithmic bevel gear and then used the corresponding relationships between the image feature points and the optical imaging geometric model to obtain the tooth surface point parameters. To determine the tooth profile, Li et al. made use of the 3D coordinates of the tooth surface points [9]. These studies laid the foundations for efficient and high-precision measurements of spiral bevel gears.

The detection of gears using machine vision methods and traditional mechanical manufacturing techniques is the primary focus of this research. The potential of a commercial triangulation sensor and a commercial confocal-chromatic sensor for the measurement of gears is studied [10]. A calculation method for gear transmission error is proposed based on the point clouds of the gear obtained by optical sensors [11]. At the same time, Wang et al. proposed a method for measuring transmission errors based on the optimal installation distance and used it to measure the transmission errors of bevel gears [12]. For measurement of the gear chamfer profile, Xu et al. introduced a method of measuring the gear chamfering profile based on a laser sensor and machine vision through two grades [13]. Simultaneously, Sun proposed two non-contact measurement methods based on machine vision and laser sensors and developed a measurement scheme for gear chamfering profile parameters based on the angle of the gear chamfering profile [14]. Fixed measuring volumes limit standard measuring methods, such as coordinate and gear measuring instruments for large gear measurements. Therefore, a model-based scanning multi-distance measurement approach for gear shape parameters is presented [15,16,17]. With the aim of solving the problems of high cost, complex maintenance, and low efficiency of existing gear detection technology, a new three-dimensional (3D) full-field optical measurement method for gears is proposed [18]. Present work puts forward a method of measuring a gear shaft radial runout with line-structured light vision. This method can measure the radial runout value and direction online for straightening the bending of the gear shaft [19]. Optical scanning technology is introduced to improve the manufacturing accuracy of polymer gears and worm gears, which provides a reliable guarantee for product quality of polymer gears and worm gears [20,21].

Current gear visual measurement methods convert gear images into gear parameters through data transformation. Compare the relevant parameters of the gear product to determine whether it is qualified. Multiple data transformations are performed in the measurement program, which will undoubtedly lengthen the measurement period. In addition, the transformation process will inevitably result in transformation errors, undoubtedly leading to large measurement errors.

As a traditional method of gear machining error measurement using machine vision, it requires calibration of the measurement system, acquisition of the gear image, grayscale conversion, noise reduction, binarization, morphological processing, Hough transform, edge extraction, and the collection of relevant measurement data. Each step of the six-step image data processing process, from grayscale to edge extraction, introduces errors and affects measurement accuracy.

The straight bevel gear measurement method proposed in this paper involves only two steps of data processing: smoothing and denoizing point clouds and registering them. The two steps are to remove the abnormal noise points in the point cloud data. Regarding the existing bevel gear visual measurement scheme, the steps of data processing are effectively reduced, thereby effectively reducing the impact of the error introduced by data processing.

There is no doubt that tooth top surface profile error is one of the most critical gear error measurement indices, which directly impacts gear pair service life. With a small profile error, the tooth top surface of the gear is relatively smooth, and the tooth surface of the gear meshing with the gear is less subject to local tooth surface wear, which extends the service life of the gear pair. Instead, it will greatly reduce the gear pair’s service life.

In summary, technology for the precision measurement of bevel gears has rapidly developed, and some research results have been achieved that have met the requirements of modern industry for the precision measurement of bevel gears. Nevertheless, existing methods of measuring the accuracy of bevel gears are not sufficiently rich. In this context, using computer graphics, information theory, and statistics, we propose a new method for measuring the accuracy of the top surface profile of a bevel gear, called the coaxial multi-ring measurement method, to increase the accuracy of bevel gear precision measurement.

## 2. Principle of Measurement

**Figure 1:**
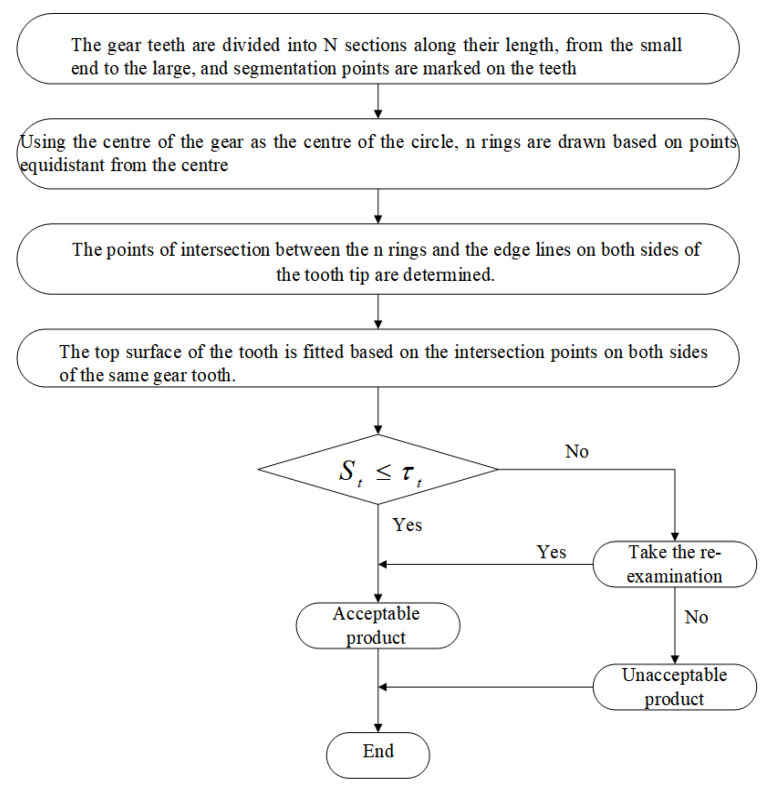
Flow chart for the coaxial multi-ring measurement method.

Our coaxial multi-ring measurement method for measuring the profile error of the top surface of the straight bevel gear tooth was developed in order to enhance the accuracy of measurement of the straight bevel gears. The method involves establishing n detection circles at equal intervals on the top surface of the straight bevel gear tooth and extracting the intersection coordinates between each detection circle and the top edge lines of all gear teeth. The intersection coordinates are then fitted with the NURBS surface theory to fit the top surface of the tooth. The surface profile error between the fitted tooth surface and the designed surface is then measured. If the surface profile error is less than a given threshold, then the product is acceptable; otherwise, the gear goes into pending re-inspection. For a further comprehensive evaluation of whether the gear is qualified, it is necessary to consider factors such as the measurement accuracy of the visual sensor and the accuracy of collecting the original point cloud data. We measure the manufacturing error of the straight bevel gear products based on the profile error on the top surface of the teeth. This process can be seen in Figure 1.

### 2.1. Step 1: Set Up Multiple Detection Circles

**Figure 2:**
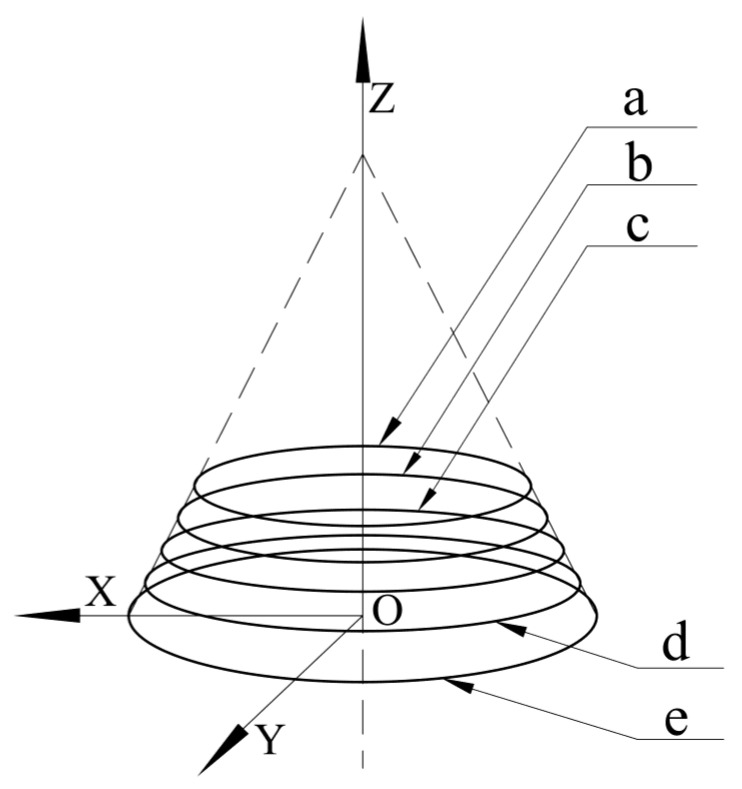
Detection circles used in the coaxial multi-ring measurement method.

1. Use the intersection point between the straight bevel gear’s central axis and its bottom surface as the origin and the bottom surface of the straight bevel gear as the O-XY plane to establish a Cartesian coordinate system. In the straight bevel gear, the Z axis is perpendicular to the bottom surface.
2. We divide the straight bevel gear teeth into n segments with equal spacing for each tooth, from the small end to the larger one.
3. We establish n detection circles by taking the distance from the center axis of the gear to all split points within the range of equal distances of 360° as the radius and using the center axis of the gear as the center. The number of detection circles will be determined based on the size of the straight bevel gear and the requirements for accurate measurement. In this case, we use an example with five detection circles;
4. Figure 2 shows the detection circles from the small end to the large end of the straight bevel gear (labeled a, b, c, d, and e).

### 2.2. Step 2: Extraction of Intersection Coordinates

**Figure 3:**
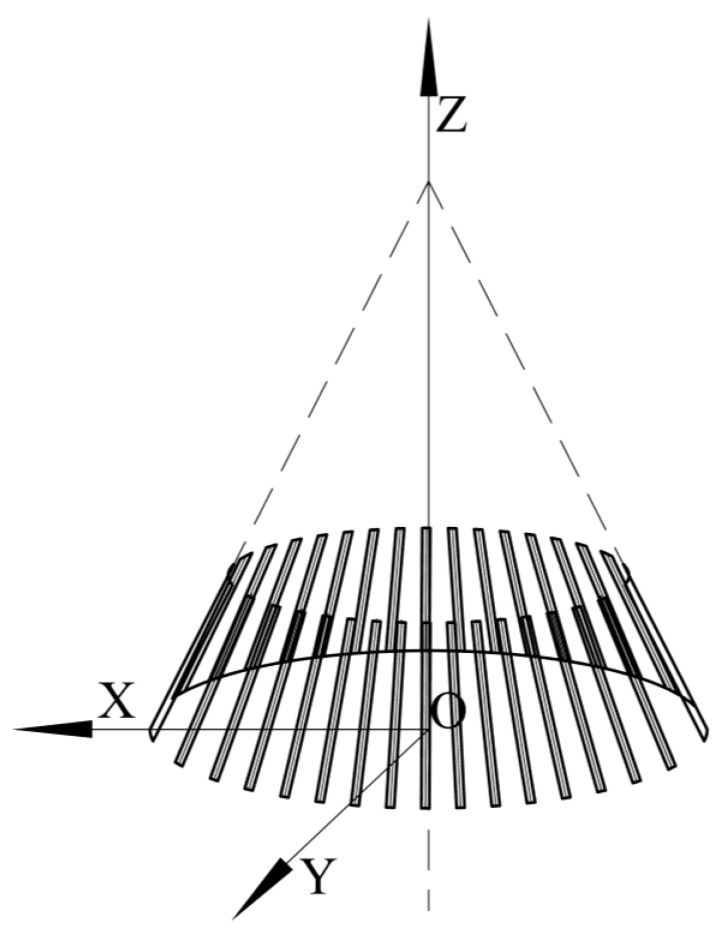
The straight bevel gear top surface model visualization.

As shown in Figure 3, we establish a Cartesian coordinate system O-XYZ for the straight bevel gear model.

**Figure 4:**
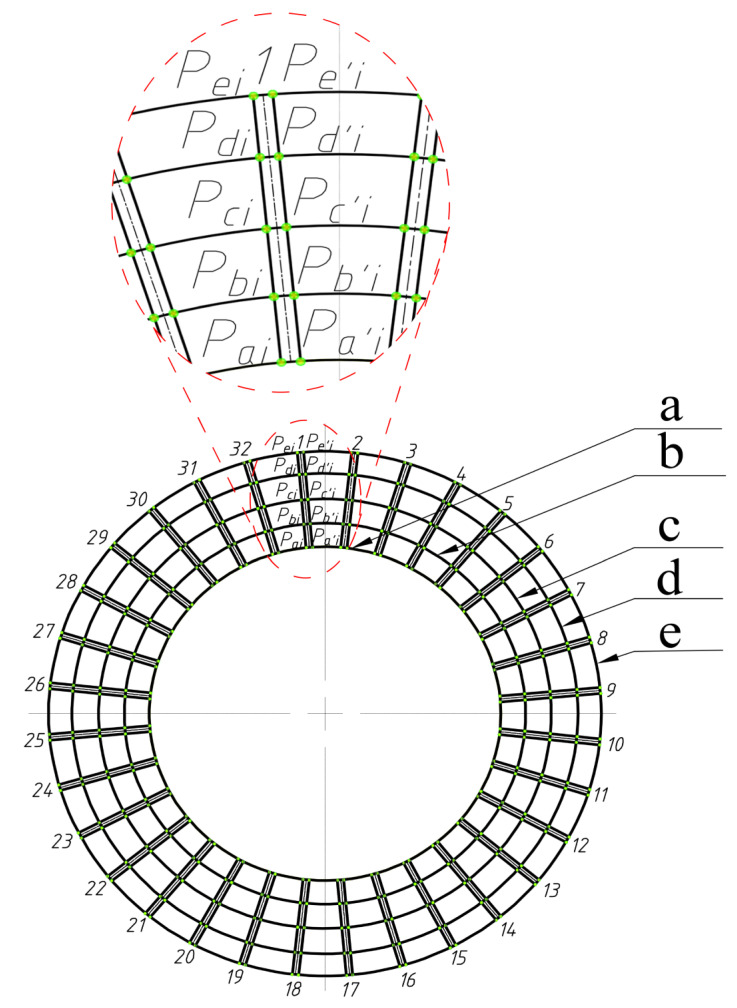
Extraction of the coordinates of the intersection points between the detection circles and the crest line.

**Figure 5:**
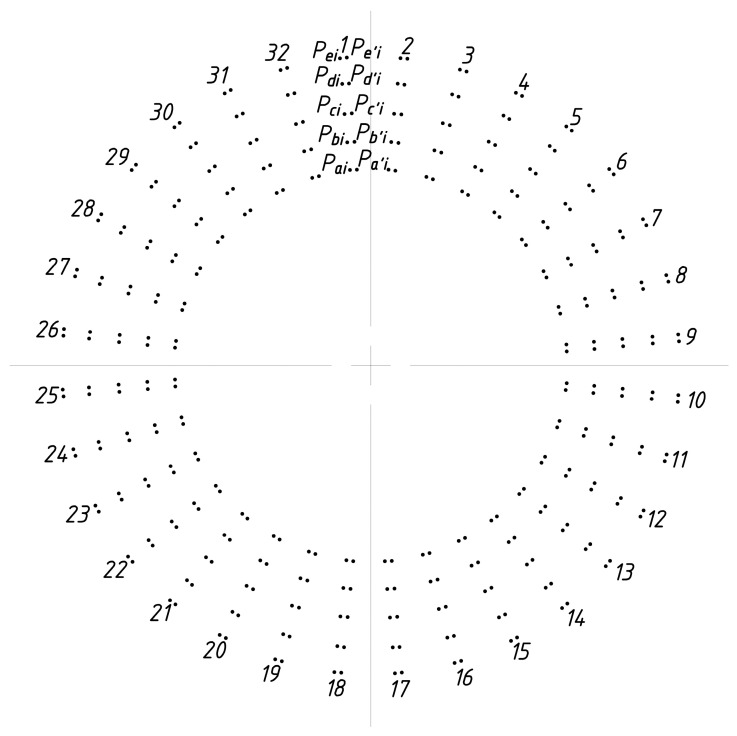
Coordinates of the intersection points between the detected circle and the tooth tip ridge line after extraction.

The gear teeth are numbered clockwise as follows, $i\in (1,n),\;n\in R^{+}$. The intersection points of circles a, b, c, d, and e with the left line of the gear teeth are referred to as $P_{ai},P_{bi},P_{ci},P_{di},P_{ei}$. The intersection points of circles a, b, c, d, and e with the right line of the gear teeth are referred to as $P_{a^{\prime }i},P_{b^{\prime }i},P_{c^{\prime }i},P_{d^{\prime }i},P_{e^{\prime }i}$. $\left(x_{ai},y_{ai},z_{ai}\right)$, $\left(x_{bi},y_{bi},z_{bi}\right)$, $\left(x_{ci},y_{ci},z_{ci}\right)$, $\left(x_{di},y_{di},z_{di}\right)$, $\left(x_{ei},y_{ei},z_{ei}\right)$ are the coordinates of the intersections between points $P_{ai},P_{bi},P_{ci},P_{di},P_{ei}$ and tooth i. $\left(x_{a^{\prime }i},y_{a^{\prime }i},z_{a^{\prime }i}\right)$, $\left(x_{b^{\prime }i},y_{b^{\prime }i},z_{b^{\prime }i}\right)$, $\left(x_{c^{\prime }i},y_{c^{\prime }i},z_{c^{\prime }i}\right)$, $\left(x_{d^{\prime }i},y_{d^{\prime }i},z_{d^{\prime }i}\right)$, $\left(x_{e^{\prime }i},y_{e^{\prime }i},z_{e^{\prime }i}\right)$ are the coordinates of the intersections between points $P_{a^{\prime }i},P_{b^{\prime }i},P_{c^{\prime }i},P_{d^{\prime }i},P_{e^{\prime }i}$ and tooth i. The coordinates of the data points in Figure 4 and Figure 5 are generated.

### 2.3. Step 3: Fitting of the Tooth Top Surface

#### 2.3.1. Cubic NURBS Curve

A cubic NURBS curve is defined as [22,23],

(1) $$p(u)=\frac{\sum_{i=0}^{n}w_{i}N_{i,3}(u)d_{i}}{\sum_{i=0}^{n}w_{i}N_{i,3}(u)}$$

where $w_{i}(0\leq i\leq n)$ is the weight factor, $d_{i}(0\leq i\leq n)$ is the control point, and $N_{i,3}(u)$ is the node vector, where,

(2) $$U=[u_{0},u_{1},\cdots ,u_{n+4}]$$

The B-spline basis function is determined by and satisfies the following recurrence relation,

(3) $$N_{i,0}(u)=\left\{\begin{array}{l} 1,u_{i}\leq u\leq u_{i+1}\\ 0,Otherwise \end{array}\right.$$

(4) $$N_{i,3}(u)=\frac{u-u_{i}}{u_{i+3}-u_{i}}N_{i,2}(u)+\frac{u_{i+3}-u}{u_{i+3}-u_{i+1}}N_{i+1,2}$$

In the case of the node vector $U=[u_{0},u_{1},\cdots ,u_{n+4}]$, the repetition degree of the nodes at both ends of the node vector is taken as four, that is,

(5) $$\left\{\begin{array}{c} u_{0}=u_{1}=u_{2}=u_{3}\\ u_{n+1}=u_{n+2}=u_{n+3}=u_{n+4} \end{array}\right.$$

#### 2.3.2. NURBS Cube Matrix Representation

For $U=[u_{0},u_{1},\cdots ,u_{n+6}]$, the following notation is used in our discussion,

(6) $$\left\{\begin{array}{l} \nabla_{i}^{1}=\nabla_{i}=u_{i+1}-u_{i}\\ \nabla_{i}^{2}=\nabla_{i}+\nabla_{i+1}=u_{i+2}-u_{i} \end{array}\right.$$

We define,

(7) $$\left\{\begin{array}{c} \nabla_{i}^{0}=0\\ t=\frac{u-u_{i}}{\nabla_{i}} \end{array}\right.$$

Based on the literature [24], the matrix expression for the i-th curve is,

(8) $$p_{i}(t)=\frac{T_{3}N_{i}H_{i}}{T_{3}N_{i}W_{i}},0\leq t\leq 1$$

### 2.4. Cubic NURBS Curve Interpolation

#### 2.4.1. Computation of the Node Vector Using Cumulative Chord Parameterization

In the definition domain, the node vector and the shape point $p_{i}\left(i=0,1,\cdots ,n\right)$ correspond to any cubic NURBS curve. The node vector of the cubic NURBS interpolation curve is $U=\left[u_{0},u_{1},\cdots ,u_{n+6}\right]$. When the parameter value $u_{i+3}(i=0,1,\cdots ,n)$ of the node vector for a given model point is $p_{i}(i=0,1,\cdots ,n)$ unknown, a parameterization method based on a cumulative chord length with high precision is used to parameterize the model value point.

(9) $$U=\left\{\begin{array}{c} u_{0}=u_{1}=u_{2}=u_{3}=0\\ u_{i+3}=\sum_{i=1}^{i}\vec{\left|p_{i}p_{i+1}\right|}/s\\ u_{n+3}=u_{n+4}=u_{n+5}=u_{n+6}=1 \end{array}\right.$$

where there are n-type value points, $p_{i}$ are the type value points, and s is the sum of chord lengths, defined as,

(10) $$s=\sum_{i=1}^{n-1}\vec{\left|p_{i}p_{i+1}\right|}$$

#### 2.4.2. Calculation of the Curve Control Points Using Boundary Tangents

The value points $p_{i}\left(i=0,1,\cdots ,n\right)$ and their corresponding (n + 3) weighting factors $w_{i}(i=0,1,2,\cdots ,n+2)$ are known. We must now determine the cubic NURBS curve $p\left(u\right)$ such that $p_{i}\left(i=0,1,2,\cdots ,n\right)$ is on the curve $p\left(u\right)$. For the node vector $U=[u_{0},u_{1},\cdots ,u_{n+6}]$, let $t=\frac{u-u_{i}}{\nabla_{i}}$ be the equation for the fitted cubic NURBS curve, and set it as the weight factor.

According to interpolation theory [25],

(11) $$\left\{\begin{array}{l} p_{0}(0)=p_{0}\\ p_{i}(1)=p_{i+1}(0)=p_{i+1}(i=0,1,2,\cdots ,n-2)\\ p_{n-1}(1)=p_{n} \end{array}\right.$$

Since $p_{n-1}(1)=p_{n}(0)$, then

(12) $$p_{i}=p_{i}(0)=\frac{\left(1,0,0,0\right)N_{i}{\left(w_{i}d_{i},w_{i+1}d_{i+1},w_{i+2}d_{i+2},w_{i+3}d_{i+3}\right)}^{T}}{\left(1,0,0,0\right)N_{i}{\left(w_{i},w_{i+1},w_{i+2},w_{i+3}\right)}^{T}},(i=0,1,\cdots ,n)$$

It then follows that,

(13) $$n_{11}w_{i}d_{i}+n_{12}w_{i+1}d_{i+1}+n_{13}w_{i+2}d_{i+2}=(n_{11}w_{i}+n_{12}w_{i+1}+n_{13}w_{i+3})p_{i},(i=0,1,\cdots ,n)$$

Assuming that,

(14) $$\left\{\begin{array}{l} a_{i}=n_{11}w_{i}\\ b_{i}=n_{12}w_{i+1}\\ c_{i}=n_{13}w_{i+2} \end{array}\right.$$

(15) $$a_{i}d_{i}+b_{i}d_{i+1}+c_{i}d_{i+2}=(a_{i}+b_{i}+c_{i})p_{i},(i=0,1,2,\cdots ,n)$$

Since this system of equations has n + 3 unknowns but only (n + 1) equations, two more equations are needed. A boundary tangent condition is often used with NURBS, which can be obtained by interpolating the endpoints of a NURBS curve.

(16) $$\left\{\begin{array}{l} p_{0}=d_{0}\\ p_{n}=d_{n+2} \end{array}\right.$$

From the boundary tangent condition [26], we have,

(17) $$\left\{\begin{array}{l} p_{0}^{\prime }(0)=\frac{3w_{1}}{w_{0}}(d_{1}-d_{0})\\ p_{n-1}^{\prime }(0)=\frac{3w_{n+1}}{w_{n+2}}(d_{n+2}-d_{n-1}) \end{array}\right.$$

(18) $$\left\{\begin{array}{l} a_{0}=\frac{3w_{1}}{w_{0}}\\ b_{0}=\frac{3w_{1}}{w_{0}}\\ e_{0}=p_{0}^{\prime }(0)\\ e_{i}=(a_{i-1}+b_{i-1}+c_{i-1})p_{i-1},(i=0,1,2,\cdots ,n+1)\\ b_{n+2}=-\frac{3w_{n+1}}{w_{n+2}}\\ c_{n+2}=-\frac{3w_{n+1}}{w_{n+2}}\\ e_{n+1}=c_{n-1}^{\prime }(1)\\ e_{n+2}=c_{n-1}^{\prime }(1) \end{array}\right.$$

As a result, we have the following system of linear equations,

(19) $$\left[\begin{array}{cccccc} a_{0} & b_{0} & & & & \\ a_{1} & b_{1} & c_{1} & & & \\ & a_{2} & b_{2} & c_{2} & & \\ & & \ddots & \ddots & \ddots & \\ & & & a_{n+1} & b_{n+1} & c_{n+1}\\ & & & & b_{n+2} & c_{n+2} \end{array}\right]\left[\begin{array}{c} d_{0}\\ d_{1}\\ d_{2}\\ \vdots \\ d_{n+1}\\ d_{n+2} \end{array}\right]=\left[\begin{array}{c} e_{0}\\ e_{1}\\ e_{2}\\ \vdots \\ e_{n+1}\\ e_{n+2} \end{array}\right]$$

This equation system can be used to establish and solve for the control vertex $d_{i},(i=0,1,2,\cdots ,n)$, and the initial weight factor for the type-valued points is one.

Thus, we define a bicubic NURBS surface as follows [27],

(20) $$p(u,v)=\frac{\sum_{i=1}^{n}\sum_{j=1}^{m}N_{i,3}(u)N_{j,3}(v)w_{ij}D_{ij}}{\sum_{i=1}^{n}\sum_{j=1}^{m}N_{i,3}(u)N_{j,3}(v)w_{ij}}$$

where

(21) $$\left\{\begin{array}{l} u,v-Vector\;node\\ D_{ij}(0\leq i\leq n,0\leq j\leq m)-Weighting\;factor\\ W_{ij}(0\leq i\leq n,0\leq j\leq m)-Weighting\;factor\\ N_{i,3}N_{j,3}-B-spline\;basis\;functions \end{array}\right.$$

Using the above fitting curve above, we take the control vertex $D_{i}$ as the type value and calculate the control vertex $D_{ij}$ along the opposite direction, and the surface can then be fitted.

**Figure 6:**
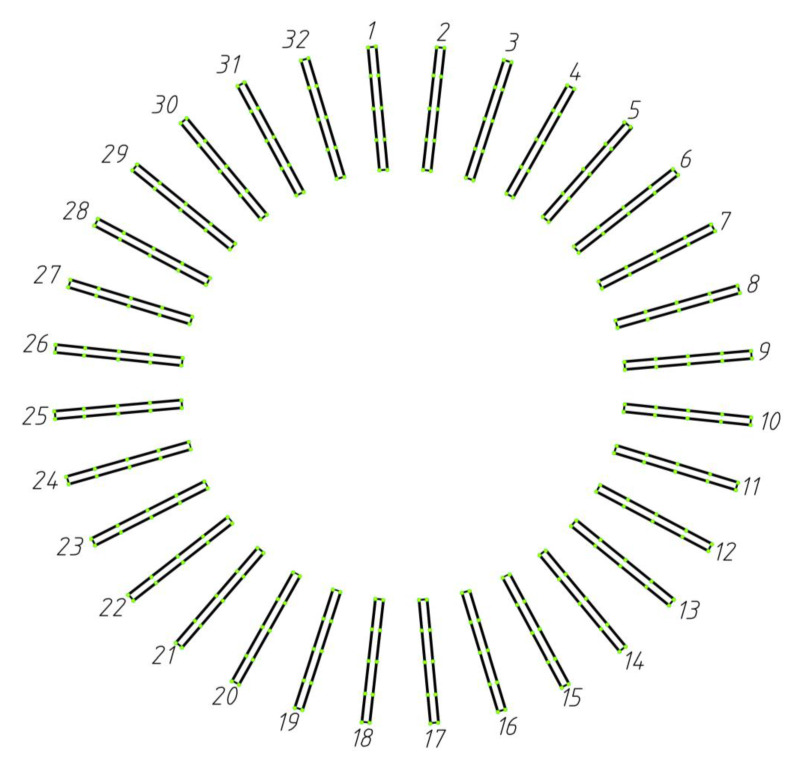
Simulation diagram of the fitted top surface of the tooth.

Based on the theoretical model in Section 2.4, the model of the top surface of the tooth in Figure 6 can be fitted by multiple calculations.

### 2.5. Measurements of the Surface Profile Deviation

**Figure 7:**
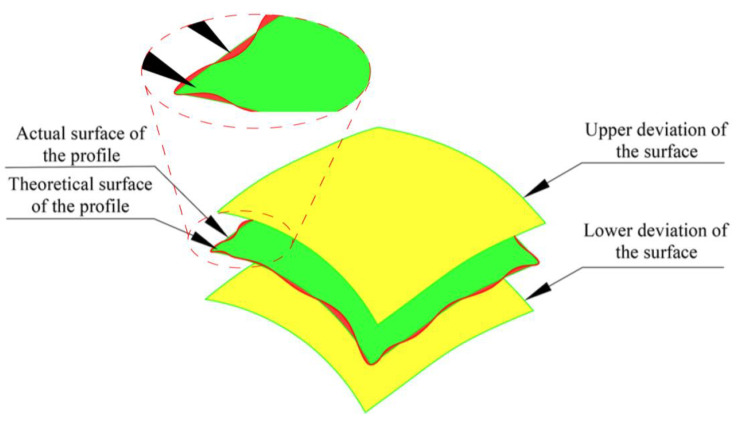
Schematic diagram of surface profile errors.

The surface profile error is the minimum distance between two theoretically equidistant surfaces containing a measured contour, as shown in Figure 7. An effective surface profile is a comprehensive tolerance that controls both the surface profile error and the line profile error of any profile on a surface.

We assume that the theoretical model point $p_{i,j}^{\prime }(x_{i,j}^{\prime },y_{i,j}^{\prime },z_{i,j}^{\prime })$ corresponds to the measurement point $q_{i,j}(x_{i,j}^{\prime \prime },y_{i,j}^{\prime \prime },z_{i,j}^{\prime \prime })$, i=1,2,⋯,A, j=1,2,⋯,B and that A and B are the numbers of measurement points in x and y. Then, the distance between $q_{i,j}$ and $p_{i,j}^{\prime }$ can be expressed as [28],

(22) $$d_{i,j}=\pm \sqrt{{\left(x_{i,j}^{\prime }-x_{i,j}^{\prime \prime }\right)}^{2}+{\left(y_{i,j}^{\prime }-y_{i,j}^{\prime \prime }\right)}^{2}+{\left(z_{i,j}^{\prime }-z_{i,j}^{\prime }\right)}^{2}}$$

And,

(23) $$\left\{\begin{array}{c} q_{i,j}>p_{i,j}^{\prime },d_{i,j}>0\\ q_{i,j}\leq p_{i,j}^{\prime },d_{i,j}\leq 0 \end{array}\right.$$

where $d_{i,j}$ represents the distance between the actual position of the measurement point and the corresponding design position. The practical significance of the verification of straight bevel gear machining accuracy is as follows: a positive value indicates that the actual surface of the tested gear deviates from the design position, and additional material is present at the machined position, while a negative value indicates that the actual surface of the tested gear is within the design surface, indicating overcutting at that point.

In view of the complexity and non-rotational symmetry of the free-form surface, the shape error is described by the surface average contour error ($S_{t}$), which is defined as [28],

(24) $$S_{t}=\frac{1}{m^{\prime }n^{\prime }}\sum_{i=1}^{m^{\prime }}\sum_{j=1}^{n^{\prime }}d_{i,j}$$

The number of points on the measured surface is ($n^{\prime }\times m^{\prime }$).

## 3. Experiments

### 3.1. Measurement Device

Optical measurements were performed with an ATOS Q 12M optical scanning system from GOM, a subsidiary of Carl Zeiss, Germany. Table 1 shows the parameters of the device [29,30].

### 3.2. Preparation

**Figure 8:**
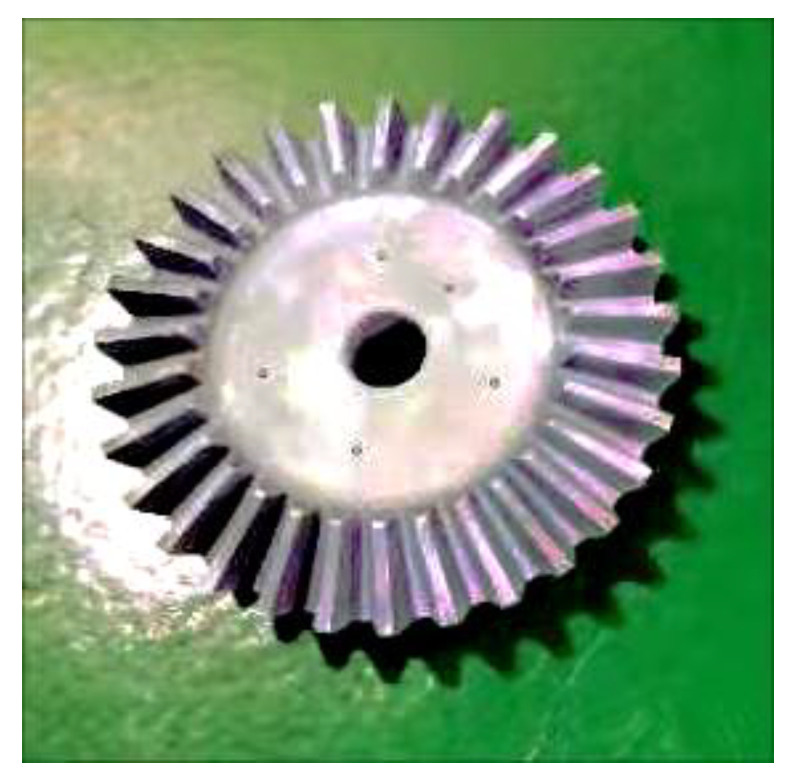
Gear to be evaluated.

5. The gear was milled using a 275 HC bevel gear milling machine. The gears were cleaned with an ultrasonic gear cleaner before testing to ensure that the surfaces were clean;
6. Multiple copies of the marker points were made and were pasted randomly onto the surface of the tested gear to improve the accuracy of stitching of the gear point cloud data;
7. Since a gear is a highly reflective component, to obtain a more complete and accurate gear point cloud, it was necessary to apply a thin layer of an imaging agent to the gear surface to reduce the adverse effects of reflection on the optical measurements. A titanium powder developer with the least impact on optical measurements was selected to achieve highly accurate gear measurements, and the size increased by 1–2 μm once sprayed [31]. The developer had a negligible impact on the overall gear size since it was large. A surface-treated straight bevel gear is shown in Figure 8, and the gear parameters are shown in Table 2.

### 3.3. Data Collection

**Figure 9:**
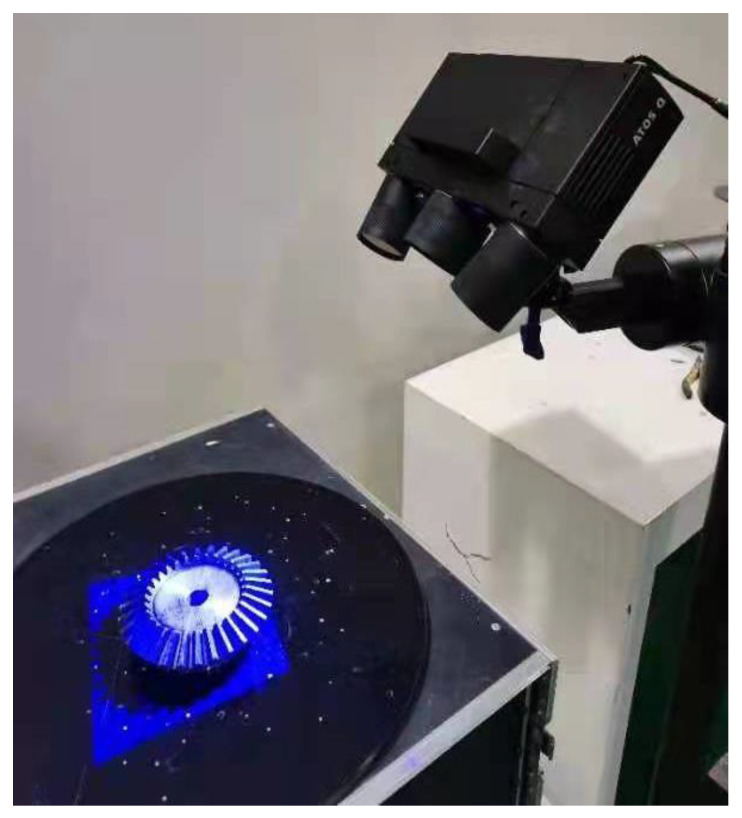
Equipment used for gear data collection.

**Figure 10:**
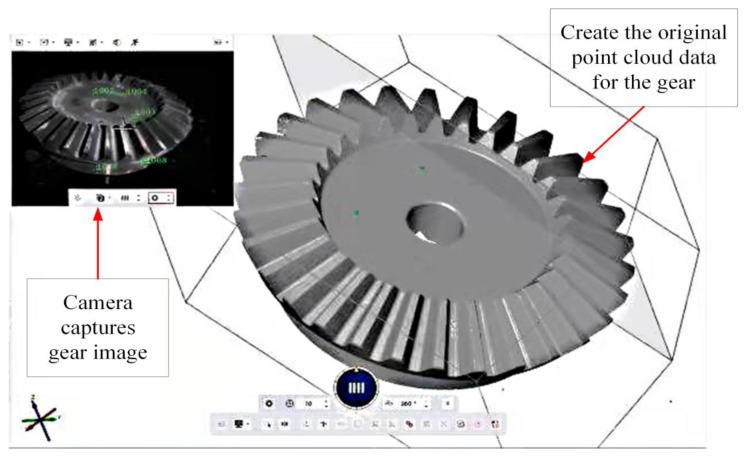
Collected gear point clouds.

We fixed the gear to the measuring table and set the table to a suitable height, as shown in Figure 9. Data acquisition of the surface-treated gears and zone-scanning of gear surfaces were carried out with the ATOS optical scanning system. By measuring the common marker points every time, the point cloud data of the gear scanned by multiple partitions was spliced with the local point cloud for the gear, giving the complete gear point cloud data model shown in Figure 10 [32].

### 3.4. Cloud-Based Data Processing

The point cloud data were smoothed by empirical modal decomposition. The gear point cloud data can be smoothed and denoized by new filters; for example, a low-pass filter can be used for smoothing, and a high-pass filter can be used for data processing.

8. Filter design

Point cloud empirical mode decomposition (EMD) can be used for various applications by developing several different filters. A filter design can be realized using the intrinsic mode function (IMF) and a margin obtained from the EMD as follows [33],

(25) $$g^{\prime }=\sum_{k=1}^{N}\eta (k)\cdot D_{k}+r_{N}$$

where $g^{\prime }$ represents the input signal; η(k) is used to control the different filter operations; $D_{k}$ is the intrinsic modal function obtained through decomposition; and $r_{N}$ is the relevant margin.

9. Smoothing and denoizing of data

Point cloud EMD involves designing filters to smooth the point cloud data. If noise is usually found in the first few IMFs, a low-pass filter is applied to eliminate the high-frequency components while keeping the low-frequency ones to remove the noise. To smooth the point cloud data, we use a low-pass filter, which is simply the margin obtained by the feature-preserving point cloud EMD, for reconstruction. In order to prevent the blurring of sharp edges and corners during the extraction of the details, the feature points in the point cloud data are extracted by existing methods in advance. In order to better reconstruct the smooth point cloud model, the normal direction is updated by the normal direction weighted average as follows [33],

(26) $$N{\left(i\right)}^{\prime }=\frac{1}{\sum_{j\in N(i)}g(p_{i})}\sum_{j\in N(j)}g(p_{i})N(i)$$

where $N{\left(i\right)}^{\prime }$ is the extreme point, $g(p_{i})$ is the corresponding input signal value, and N(i) is the point adjacent to $P_{i}$.

**Figure 11:**
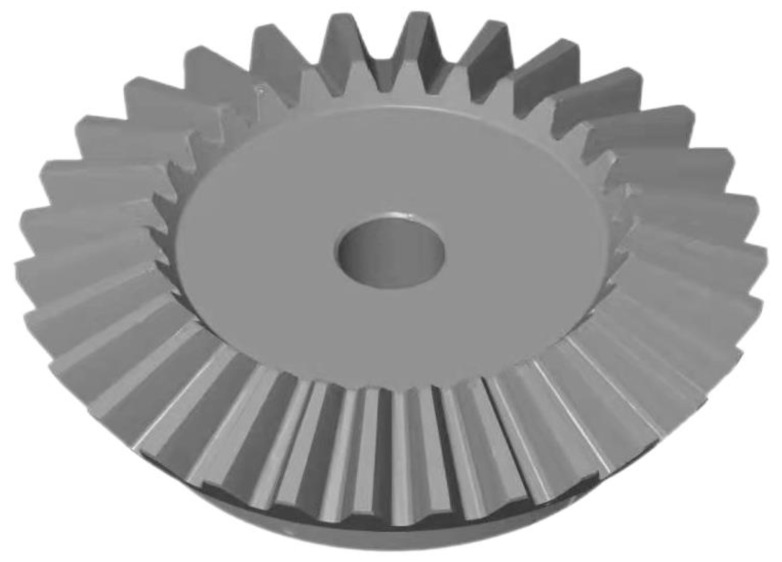
Preprocessed point clouds.

After smoothing and denoizing the point cloud data through the filter in Section 3.4, the gear point cloud data is obtained, as shown in Figure 11.

### 3.5. Implementation of the Proposed Straight Bevel Gear Coaxial Multi-Ring Measurement System

**Figure 12:**
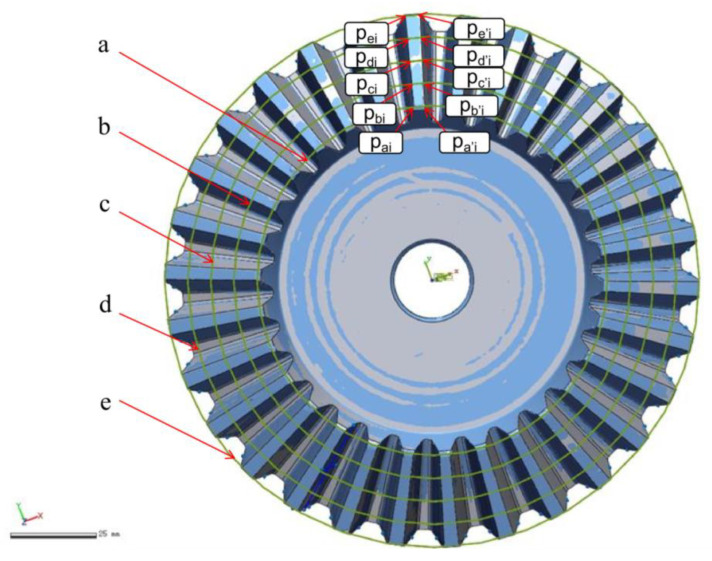
Validation of the proposed coaxial multi-ring measurement method.

As explained in Section 2.1, n coaxial detection circles were established on the straight bevel gear model. Five detection circles are given here as examples and are marked a, b, c, d, and e. From Section 2.2, it can be seen that the detection circle and additional ridgelines intersect at points $P_{ai},P_{bi},P_{ci},P_{di},P_{ei}$ and $P_{a^{\prime }i},P_{b^{\prime }i},P_{c^{\prime }i},P_{d^{\prime }i},P_{e^{\prime }i}$, respectively, as shown in Figure 12.

**Figure 13:**
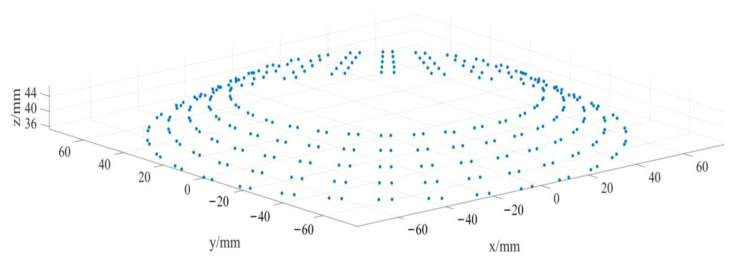
Calculated gear point coordinates.

**Figure 14:**
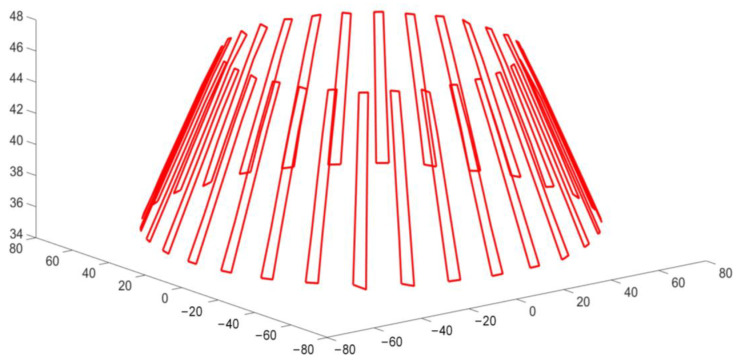
Fitting the tooth surface’s perspective projection.

We determined the coordinates of the intersection points $P_{ai},P_{bi},P_{ci},P_{di},P_{ei}$ and $P_{a^{\prime }i},P_{b^{\prime }i},P_{c^{\prime }i},P_{d^{\prime }i},P_{e^{\prime }i}$, based on Figure 12 and calculated their positions in 3D space through MATLAB, which were used to generate Figure 13. Figure 14 shows the result of fitting the top surface of the gear tooth using the NURBS surface theory, as described in Section 2.3.

## 4. Results and Analysis

**Figure 15:**
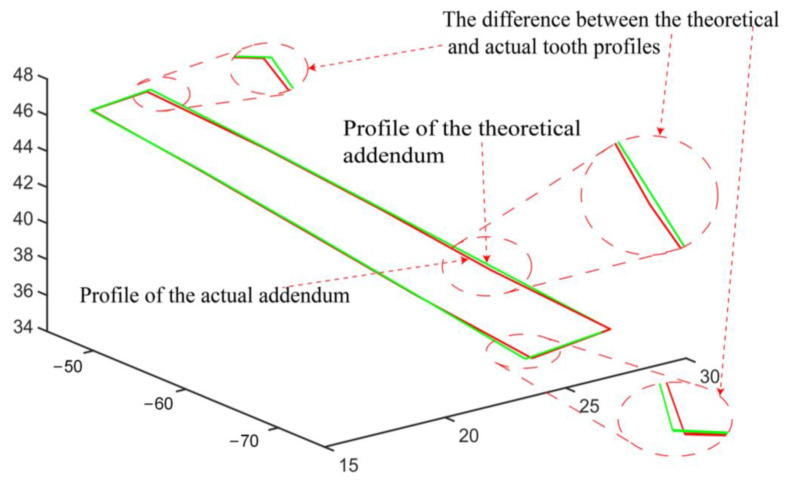
Top surface profile deviation for one gear tooth.

**Figure 16:**
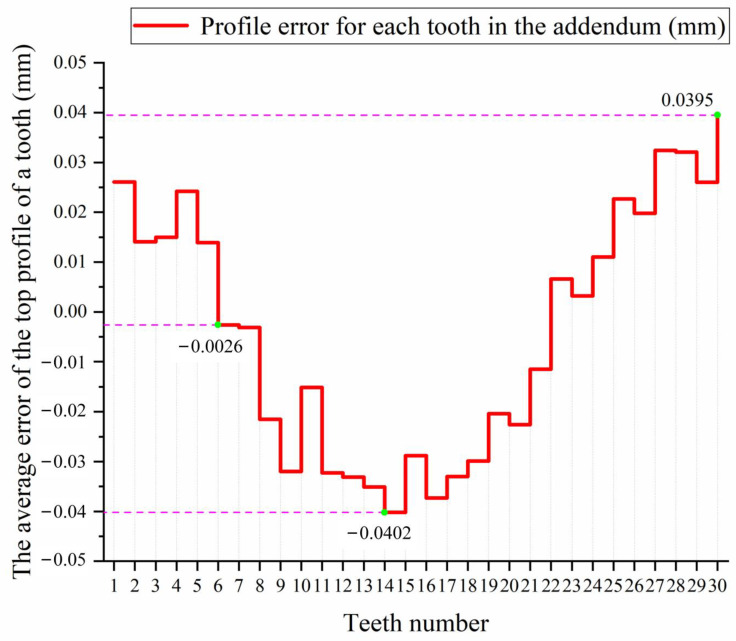
Error in the mean profile of each tooth surface.

We can compare the fitted actual top surface of the tooth with the theoretical surface to find the error in the contact profile. The difference between the theoretical top surface of a gear tooth and the actual surface is shown in Figure 15. Using the surface profile measurement method in Section 2.5, we measured the surface profile error for each of the 30 gear teeth and then calculated the average value of the surface profile error for each tooth. As a consequence of the statistical principle, the average surface profile error of the tooth top surface over the 30 teeth of the gear is statistically analyzed, as shown in Figure 16.

As can be seen in Figure 16, the average surface profile error for the top surface of the gear teeth is within the range of −0.0402 to 0.0395 mm. The figure shows the top surface of tooth 14, which had the largest average surface profile error of −0.0402 mm. The minimum average surface profile error was observed for the top surface of the sixth gear tooth, with a value of 0.0026 mm. The average profile error for all the teeth of this gear was −0.0373 mm.

As a result of this measurement method, the average surface profile of the top surface of the gear tooth can be used to evaluate whether or not the gear is an acceptable product. If the average profile deviation in the top surface of all the gear teeth is less than a given threshold τ_t_ (which is set based on the product use requirements), then the gear is an acceptable product; otherwise, the gear goes into pending re-inspection. For a further comprehensive evaluation of whether the gear is qualified, it is necessary to consider factors such as the measurement accuracy of the visual sensor and the accuracy of collecting the original point cloud data.

## 5. Conclusions

A combination of computer vision technology, computer graphics, error theory, and statistical principles is used to measure the top surface error of the straight bevel gears. The surface profile error of the tooth top surface is used as the detection index and forms the basis for judging whether or not the gear is an acceptable product. This is mainly achieved by establishing multiple detection circles on the top surface of the gear tooth and then obtaining the coordinates of the intersection points between the detection circles and the edge lines for all gear teeth. The intersection coordinates are fitted to the tooth top surface based on the NURBS surface theory. We then measure whether the surface profile error between the fitted and designed top surfaces exceeds a given threshold, which is determined by the product use requirements; if it is less than the threshold, the product is acceptable.

Our measurement method was verified, and it was found that the profile error in the top surface of the straight bevel gear tooth could be fully measured, and the machining error of the gear could be accurately determined. The principle and process of our proposed method represent a new approach to gear error measurement technology and contribute to enriching the current range of gear error measurement methods, broadening the scope of gear error measurement, and promoting the development of measurement techniques for modern gears.

## Figures and Tables

**Table 1:** Parameters of the measurement system.

Lens Parameter	Data
Camera Pixel	2 × 3296 × 2472 Pixels 0.2 mm–10 m (paired photogrammetry)
Scan range	
Camera model	MV170
Measurement volume	170 × 130 × 130 mm
Measurement Accuracy	0.009 mm
Point spacing	0.03–0.12 mm
Working distance	490 mm
Projected light source	Structured blue light (LED)
Operating Temperature	5–40 °C

**Table 2:** Parameters of the measured gear.

Parameter	Value
Number of teeth	30 5
Module/mm	
Reference diameter/mm	75
Cone distance/mm	83.853
Tip angle/°	49.12
Root angle/°	40.88
Reference cone angle/°	45
Tooth width/mm	27.951

## Data Availability

Not applicable.
